# An Efficient Steady-State Analysis Method for Large Boolean Networks with High Maximum Node Connectivity

**DOI:** 10.1371/journal.pone.0145734

**Published:** 2015-12-30

**Authors:** Changki Hong, Jeewon Hwang, Kwang-Hyun Cho, Insik Shin

**Affiliations:** 1 School of Computing, KAIST, Daejeon, Korea; 2 Department of Bio and Brain Engineering, KAIST, Korea; King’s College London, UNITED KINGDOM

## Abstract

Boolean networks have been widely used to model biological processes lacking detailed kinetic information. Despite their simplicity, Boolean network dynamics can still capture some important features of biological systems such as stable cell phenotypes represented by steady states. For small models, steady states can be determined through exhaustive enumeration of all state transitions. As the number of nodes increases, however, the state space grows exponentially thus making it difficult to find steady states. Over the last several decades, many studies have addressed how to handle such a state space explosion. Recently, increasing attention has been paid to a satisfiability solving algorithm due to its potential scalability to handle large networks. Meanwhile, there still lies a problem in the case of large models with high maximum node connectivity where the satisfiability solving algorithm is known to be computationally intractable. To address the problem, this paper presents a new partitioning-based method that breaks down a given network into smaller subnetworks. Steady states of each subnetworks are identified by independently applying the satisfiability solving algorithm. Then, they are combined to construct the steady states of the overall network. To efficiently apply the satisfiability solving algorithm to each subnetwork, it is crucial to find the best partition of the network. In this paper, we propose a method that divides each subnetwork to be smallest in size and lowest in maximum node connectivity. This minimizes the total cost of finding all steady states in entire subnetworks. The proposed algorithm is compared with others for steady states identification through a number of simulations on both published small models and randomly generated large models with differing maximum node connectivities. The simulation results show that our method can scale up to several hundreds of nodes even for Boolean networks with high maximum node connectivity. The algorithm is implemented and available at http://cps.kaist.ac.kr/∼ckhong/tools/download/PAD.tar.gz.

## Introduction

Modeling of biological systems as a network of interacting components has received increasing attention in various areas, such as computational and systems biology since it allows to analyze biological phenomena systematically at various scales including molecular and cellular levels [1]. Boolean networks (BNs) have been widely used among various network models because BNs are relatively simple and efficient to model large networks [2–4]. The BN is a discrete model of biological system that comprises of a number of nodes and corresponding update rules. Each node represents a gene and takes on a value of 1 or 0, meaning that the gene is expressed or unexpressed, respectively. Each update rule represents interactions between genes. The state of a gene at a given time step is determined by its update rule and the state of its input genes at the previous time step. In synchronous BNs, the states of all nodes are updated simultaneously at each time step, and it directly induces global state transitions. An important characteristic of BNs is that any sequence of consecutive global state transitions eventually converges to either a single state (*i.e., steady state*) or a cycle of states (*i.e., cyclic attractors*).

It is important to identify steady states to have a proper understanding of biological systems because steady states often convey biological implications. Specifically, steady states capture long-term behaviors of biological systems: they are closely related to different cell types or states (*e.g.,* growth, differentiation, and apoptosis) [5–7]. This motivated several meaningful case studies of using steady-state analysis. For example, the identification of steady states has been playing a crucial role in treatment of various human cancers such as breast cancer, and leukemia [8, 9]. Additionally, the steady-state analysis can successfully explain the flower morphogenesis of Arabidopsis thaliana [10], the differentiation process of T-helper cells [11], the mechanism of T cell receptor signaling [12], the cell cycles of yeast types [13, 14], and the express patterns of Drosophila melanogaster segment polarity genes [15]. Theoretically, steady states can be detected by exhaustively exploring all the global states of a BN. However, it becomes too memory- and time-consuming even for a small BN with *n* nodes since 2^n^ global states need to be examined in total. Indeed, it has been proved that the problem of finding fixed points in a Boolean network is NP-hard [16]. Hence, it is essential to develop an efficient algorithm to detect steady states while avoiding such state space explosion.

Algorithms for the problem of finding steady states have been extensively studied in the past decade [17–30]. A common approach is to convert explicit state transitions to implicit representations: decision diagram (DD), and propositional logic formula (SAT). In algorithms based on DD [18–21], a DD represents a Boolean update rule. Then, by combining the DD representations of all the Boolean update rules, the problem of finding steady states becomes a search problem in the larger DD. This limits DD-based algorithms to small BNs with about 100 nodes. The SAT-based algorithms [22–25], however, do not suffer from the potential space explosion of DDs, but most of them have focused on large BNs with low maximum node connectivity (*i.e.,* maximum indegree, *K*). For example, Tamura *et al.* [22] proved that the problem of detecting steady states of a BN with *K* can be transformed to (*K* + 1)-SAT problem. Those algorithms take advantage of modern success of SAT solvers [31], which enable to scale up to hundreds of nodes for BNs with *K* < 2 [24]. However, in case of BNs with higher *K*s, a state explosion still occurs because no efficient SAT solvers are known for the (*K* + 1)-SAT problem with *K* ≥ 2, which is a well-known NP-complete problem [32]. This limits the SAT-based algorithms to Boolean networks with low *K*s only.

To expand the range of feasible BNs, partitioning-based steady states detection algorithms have been published recently [25, 33]. The key idea of those approaches is to reduce the basic unit of steady-state analysis. For example, Guo *et al.* [25] partition the given network into smaller blocks based on a strongly connected component (SCC). Steady states are independently detected in each block by applying a SAT solver, and then combined to construct the steady states of the original BN. Therefore they achieved a better scalability on randomly generated BNs with *K* ≤ 3. The scalability gained in principle is, however, unlikely to happen in the models of realistic biological processes. This is because *K* of these models is known as orders of magnitude higher than the average indegree [34]. For BNs with such high *K*, the size of the largest SCC is too large to be analyzed within a reasonable timing. Several studies have discovered that the currently available protein interaction networks have a SCC connecting the vast majority of the proteins [35–37]. It is also reported that the currently available metabolic and signal transduction networks are connected, with 50 to 60% of the nodes forming the largest SCC [38, 39]. Thus, the partitioning strategy based on SCC is not suitable in the real world, and so a better partitioning method is necessary.

Then, a natural question is what the smallest possible unit for partitioning is. In this manuscript, we aim to discover the smallest partition of the network to utilize a SAT solver in the most efficient way. To this end, this paper proposes an optimal partitioning algorithm based on the *minimum essential block* (MEB). Then, we build upon the idea of the MEB-based network partition into subnetworks. Each subnetwork is guaranteed to be the smallest both in the size (*i.e.,* *n*) and the maximum indegree (*i.e.,* *K*) such that any deletion of nodes or edges from the subnetwork cannot correctly determine the steady states of the network. Thus, the proposed MEB-based partition guarantees to maximize the performance of a SAT solver while ensuring to determine all the steady states reliably. We implemented an experimental tool and compared with other state-of-the-art methods [24, 25]. For the models of real biological processes acquired from literature [9–15], the runtime of the proposed algorithm performs about three times better than others in average. Since the models consist of several tens of nodes only, we randomly generated larger networks to evaluate how well our approach scales up. In comparison to the other methods, our method performs favorably on the large network with high *K*, and reliably finds all steady states even for networks with up to *n* = 1000 and *K* = 5.

The rest of this paper is organized as follows: The Methods section describes the model, definitions and the proposed algorithm. In the Results section, we demonstrate the correctness and efficiency of our method. The Discussion section sums up the results of the study.

## Methods

### The Boolean network and attractors

A *Boolean network* (BN) *G* = ⟨*V*, *F*⟩ consists of a set *V* of *n* nodes and a set *F* of Boolean update rules, where

$$\begin{array}{ccc} V & = & \left\{v_{1},v_{2},...,v_{n}\right\}\\ F & = & \{f_{1},f_{2},...,f_{n}\}. \end{array}$$

Each node *v*_*i*_ ∈ *V* has its associated state *s*_*i*_(*t*)∈{0, 1} at any time instant *t* and a Boolean update rule *f*_*i*_. The Boolean update rule *f*_*i*_: {0, 1}^*k*_*i*_^ → {0, 1} determines the state value of *v*_*i*_ (*i.e.,* *s*_*i*_(*t*)) according to the state of *k*_*i*_ neighbor nodes incoming to *v*_*i*_ at previous time instant *t* − 1. Here, *k*_*i*_ is the number of inputs for *v*_*i*_, and called *indegree* of *v*_*i*_. The maximum indegree of a Boolean network is defined as *K* = max{*k*_*i*_}. The states of all nodes (*i.e.,* global state) are updated simultaneously at each time instant. We denote the global state of the Boolean network at time *t* by a vector *S*(*t*) = ⟨*s*_1_(*t*), *s*_2_(*t*), …, *s*_*n*_(*t*)⟩. Hence, the synchronous updating of all nodes directly induces global state transitions as follows:

$$\begin{array}{c} F(S(t))=S(t+1) \end{array}$$

The state transition represents the dynamics of the network. One of the main characteristics of Boolean network dynamics is that for any initial state condition, the system eventually converges to *attractors*. Consecutive global state transitions *S*(*t*), *S*(*t* + 1), …, *S*(*t* + *p*) are called an attractor with period *p* if *S*(*t*) = *S*(*t* + *p*). An attractor with period *p* = 1 is called a *steady state* (*i.e.,* a *fixed point*), and an attractor with a period *p* > 1 is called a *cyclic attractor*.

### An efficient steady-state identification method through partitioning

The state space of a BN grows exponentially with the number of nodes in the network. It becomes more complex as the maximum indegree increases. In this paper, we divide the given network into multiple smaller subnetworks to decrease the computation complexity. We then independently analyze each subnetwork to find *local* steady states by applying a SAT solver. Finally, we compose the local steady states to construct the steady states of the overall network. Note that the steady states detected in each subnetwork are called local steady states to distinguish them from the steady states of the given original BN.

#### Intuitive idea

The steady states identified through partitioning should be identical to the steady states detected directly from the original BN. This and the following section present how our partition-based method can be applied to identify steady states in a complete and correct manner. For ease of discussion, we first describe an intuitive idea of how we divide a network into two subnetworks and how we combine the steady states of them to construct the steady states of the overall network. This discussion will be extended to include the case of multiple subnetworks.

**Fig 1:**
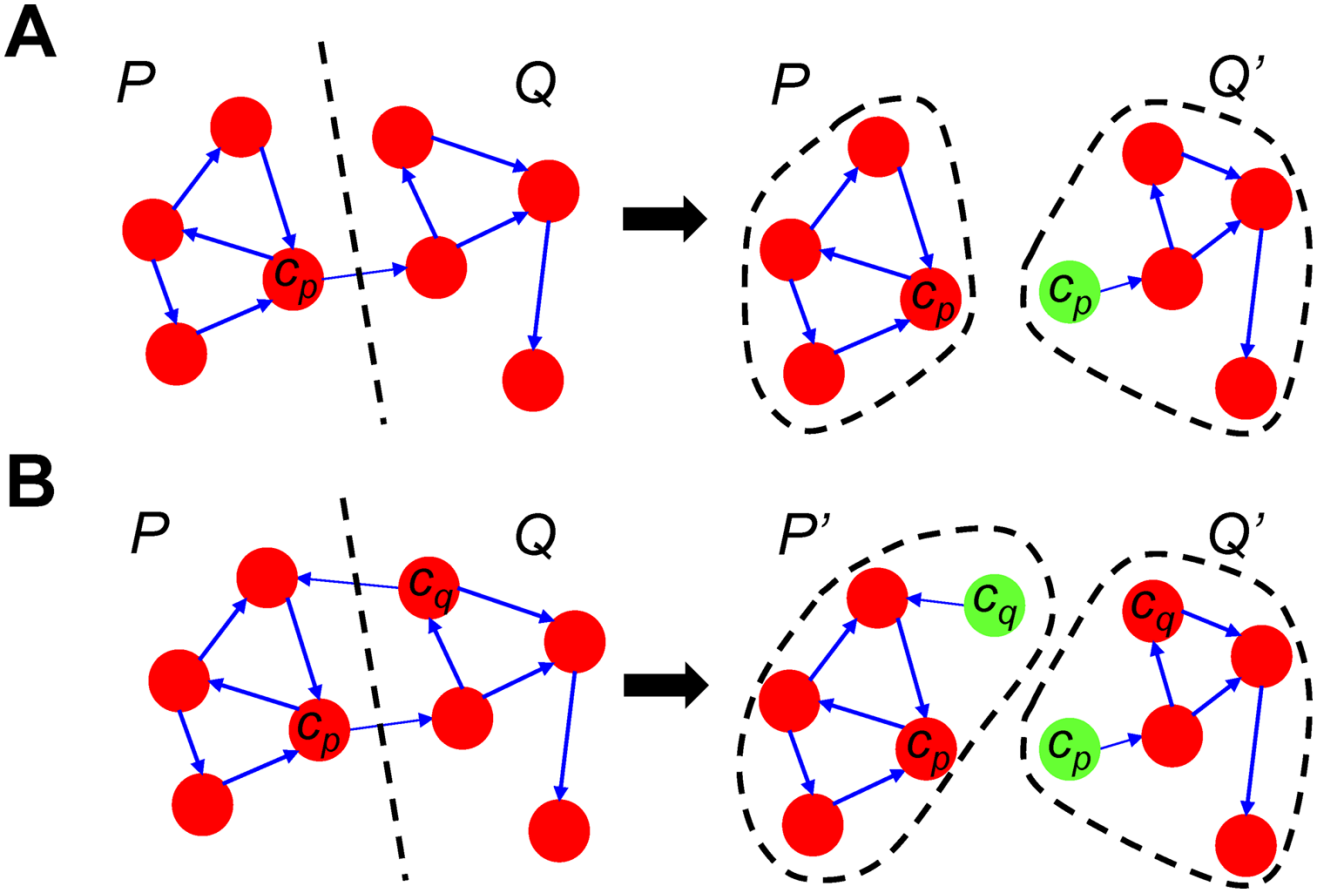
An intuitive idea of partitiong a Boolean network into two blocks and composing steady states found independently. A: A Boolean network *G* can be partitioned into two blocks *P* and *Q* which are connected by *c*_*p*_. Then, we construct two subnetworks *P* and *Q*′ where *Q*′ = *Q*∪{*c*_*p*_}, and *c*_*p*_ is used to combine the local steady states found in each subnetwork. B: When there also exists a set of edges from *c*_*q*_ of *Q*, we construct two subnetworks *P*′ and *Q*′ where *P*′ = *P*∪{*c*_*q*_} and *Q*′ = *Q*∪{*c*_*p*_}. Both *c*_*p*_ and *c*_*q*_ are used to combine the steady states of subnetworks.

Let us assume that the given BN *G* is partitioned into two blocks *P* and *Q* as shown in Fig 1A. Block *Q* depends on block *P* through an out-going edge from node *c*_*p*_. Thus, state transitions of *Q* are regulated by the values of *c*_*p*_ at each time instant. We define such *c*_*p*_ as a control node of *Q*. If there exists a steady state of *G*, say *a*, *P* will eventually converge to *a*_*p*_ (*i.e.,* a local steady state of *P*, which comprises the part of the steady state *a* of *G*) at some time instant *t*_*k*_ regardless of *Q*. From the time instant *t*_*k*_, the *fixed* value of *c*_*p*_ of *a*_*p*_ keeps controlling state transitions of *Q*, then *Q* also arives at *a*_*q*_ (*i.e.,* a local steady state of *Q*, which comprises the part of the steady state *a* of *G*). Finally, the composition of *a*_*p*_ and *a*_*q*_ can safely construct *a*.

Based on the aformentioned observations, we present two-block partitioning and composing methods that correctly determine steady states of the given network *G*. Once *G* is partitioned into two blocks *P* and *Q*, we construct two subnetworks based on these blocks. Here, it is worth noting that *P* can be independently analyzed to find its local steady states regardless of *Q*. Block *Q*, however, cannot be correctly analyzed by itself since it depends on a control input (*i.e.,* *c*_*p*_) from outside. We thus propose to construct a new subnetwork *Q*′ for block *Q* such that *Q*′ = *Q*∪{*c*_*p*_}. For the steady-state analysis of *Q*′, the value of *c*_*p*_ is assumed to be fixed to 0 or 1, and controls state transitions of *Q*. We then join each local steady state of *P* with that of *Q*′ by using the state of *c*_*p*_ as a *glue* between them. That is, we compose the two local steady states into the steady state for *G* if *c*_*p*_ has the same value in both subnetworks.

In the case that there also exists a set of out-going edges from node *c*_*q*_ of *Q* as shown in Fig 1B, we construct two subnetworks *P*′ and *Q*′, where *P*′ = *P*∪{*c*_*q*_} and *Q*′ = *Q*∪{*c*_*p*_}. For each subnetwork, local steady states are computed under the assumption that the value of its input is fixed and controls state transitions of the subnetwork. Thus, composing two local steady states that have the same values of *c*_*p*_ and *c*_*q*_ in both subnetworks can reliably construct the steady states.

#### Partitioning and correctness

The two-block partitioning and composing methods presented in the previous section can be extended towards partitioning of a BN into multiple blocks. Let us consider the given BN *G* = ⟨*V*, *F*⟩ with *n* nodes, and *V* is partitioned into *m*-number of blocks as follows:

$$\begin{array}{c} V=\left\{v_{1},v_{2},...,v_{n}\right\}=V_{1}\cup V_{2}\cup ...\cup V_{m} \end{array}$$

where *V*_*i*_ is a proper subset of *V*, *V*_*i*_∩*V*_*j*_ is empty for *i* ≠ *j*. Each block *V*_*i*_ has incoming edges from outside of the block, and the source nodes of these edges can be interpreted as inputs for each block. Let us denote the set of inputs of the block *V*_*i*_ as *C*_*i*_. Then, the subnetwork *G*_*i*_ is constructed to be used to reveal local steady states as follows:

$$\begin{array}{c} G_{i}=\left\langle V_{i}\cup C_{i},F_{i}\right\rangle \end{array}$$

where *F*_*i*_ is a set of Boolean update rules associated to the nodes of *V*_*i*_. It is worth noting that the Boolean update rules for nodes in *C*_*i*_ are ignored since they are regarded as inputs to *V*_*i*_. But, a fixed state value (*i.e.,* 0 or 1) for each node in *C*_*i*_ controls state transitions of the block *V*_*i*_, and thus input nodes are also called as control nodes.

The proposed composing method can also be generalized to the cases of combining local steady states of multiple subnetworks. Before we describe the composition procedure, we define *SV*(*a*_*k*_, *T*) as a subvector extracted from a steady-state vector, *a*_*k*_, according to a set of target output variables, *T*. For example, let us assume that a steady state vector, *a*_*k*_ = ⟨0, 1, 0, 1, 1⟩, is ordered as (*v*_1_, *v*_2_, *v*_3_, *v*_4_, *v*_5_). The subvector extracted from *a*_*k*_ with respect to the targets, {*v*_2_, *v*_3_, *v*_4_}, (*i.e.,* *SV*(⟨0, 1, 0, 1, 1⟩, {*v*_2_, *v*_3_, *v*_4_})) is thus ⟨1, 0, 1⟩. Then, we define the composition of two local steady states from distinct subnetworks. Let us assume that *G*_1_ = ⟨*V*_1_∪*C*_1_, *F*_1_⟩ and *G*_2_ = ⟨*V*_2_∪*C*_2_, *F*_2_⟩ are the two subnetworks, and

$$\begin{array}{ccc} V_{1}\cup C_{1} & = & \left\{v_{1},...,v_{k},v_{k_{1}+1},...,v_{k_{1}+l}\right\}\\ V_{2}\cup C_{2} & = & \left\{v_{1},...,v_{k},v_{k_{2}+1},...,v_{k_{2}+m}\right\} \end{array}$$

where *v*_1_, …, *v*_*k*_ are the common components in two subnetworks. If two local steady states (*e.g.,* *a*_1_ and *a*_2_ for *G*_1_ and *G*_2_, respectively) meet the following condition,

$$\begin{array}{c} SV\left(a_{1},\left\{v_{1},...,v_{k}\right\}\right)=SV\left(a_{2},\left\{v_{1},...,v_{k}\right\}\right) \end{array}$$

then *a*_1_ and *a*_2_ can be composed (*i.e.,* *a*_1_ × *a*_2_) as follows:

$$\begin{array}{c} a_{1}\times a_{2}=a_{1}+SV\left(a_{2},\left\{v_{k_{2}+1},...,v_{k_{2}+m}\right\}\right) \end{array}$$

where + operator represents the vector concatenation. All the local steady states (*i.e.,* *a*_*i*_ ∈ *A*_1_ and *a*_*j*_ ∈ *A*_2_) satisfying the condition are composed (*i.e.,* *a*_*i*_ × *a*_*j*_) as described above. The resulting intermediate set of combined local steady states is then composed with local steady states of the next subnetworks in the same manner until the last subnetwork is reached. The entire procedure guarantees the steady states identified through partitioning to be identical to the steady states detected directly from the original BN (for a formal proof, see S1 Text).

**Fig 2:**
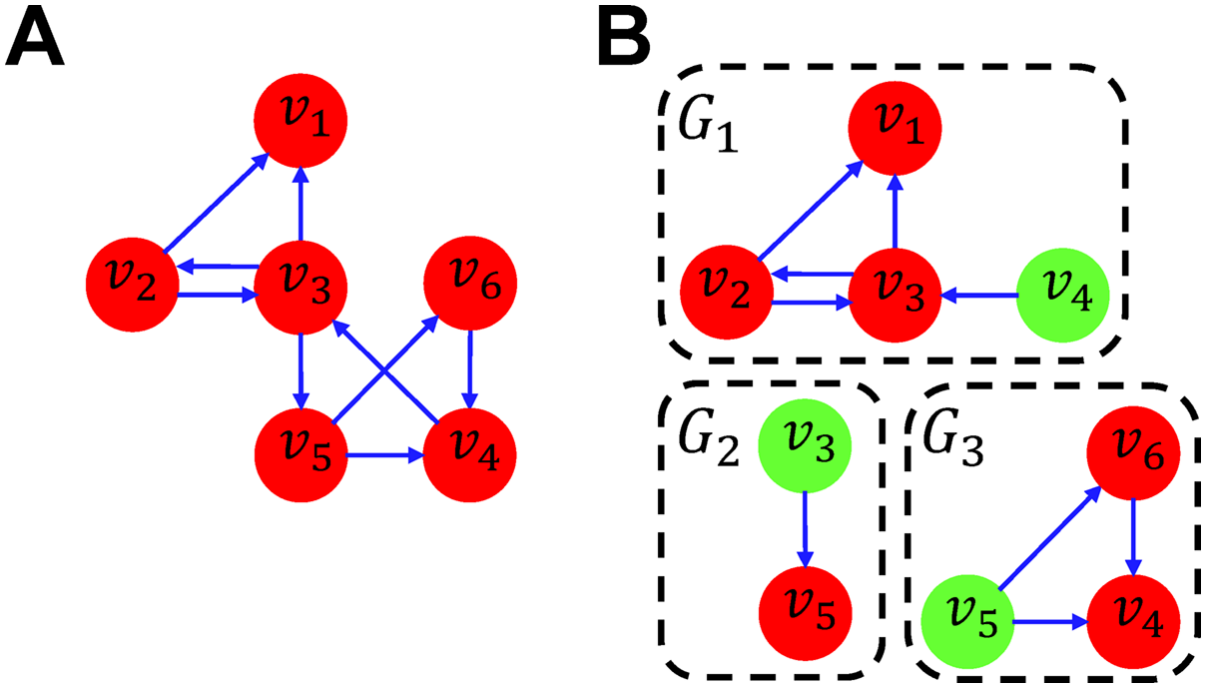
A division into three subnetworks. A: A Boolean network model *G*. B: A division into three subnetworks (*e.g.,* *G*_1_, *G*_2_, and *G*_3_). Input nodes are depicted as green nodes.

As an example, let us consider that a BN *G* = ⟨*V*, *F*⟩ with six nodes in Fig 2A, where the Boolean update rule *F* consists of six corresponding Boolean update rules as follows:

$$\begin{array}{ccc} f_{1}:s_{1}\left(t+1\right) & = & s_{2}\left(t\right)\wedge s_{3}\left(t\right)\\ f_{2}:s_{2}\left(t+1\right) & = & s_{3}\left(t\right)\\ f_{3}:s_{3}\left(t+1\right) & = & s_{2}\left(t\right)\vee s_{4}\left(t\right)\\ f_{4}:s_{4}\left(t+1\right) & = & s_{5}\left(t\right)↔s_{6}\left(t\right)\\ f_{5}:s_{5}\left(t+1\right) & = & s_{3}\left(t\right)\\ f_{6}:s_{6}\left(t+1\right) & = & \neg s_{5}\left(t\right) \end{array}$$

where ∧, ∨, ↔, ¬ denote logical AND, OR, EQUIVALENCE, NOT operations, respectively. Let us assume that *V* is partitioned into three blocks (*i.e.,* *V* = *V*_1_∪*V*_2_∪*V*_3_) as shown in Fig 2B as red nodes:

$$\begin{array}{c} V_{1}=\left\{v_{1},v_{2},v_{3}\right\},V_{2}=\left\{v_{5}\right\},V_{3}=\left\{v_{4},v_{6}\right\}. \end{array}$$

Then, their inputs are *C*_1_ = {*v*_4_}, *C*_2_ = {*v*_3_} and *C*_3_ = {*v*_5_}. Finally, three subnetworks (*i.e.,* *G*_1_, *G*_2_ and *G*_3_) can be constructed as follows:

$$\begin{array}{c} G_{1}=\left\langle V_{1}\cup C_{1},F_{1}\right\rangle ,G_{2}=\left\langle V_{2}\cup C_{2},F_{2}\right\rangle ,G_{3}=\left\langle V_{3}\cup C_{3},F_{3}\right\rangle \end{array}$$

$$\begin{array}{ccc} F_{1} & : & \left\{ \begin{array}{c} s_{1}\left(t+1\right)=s_{2}\left(t\right)\wedge s_{3}\left(t\right)\\ s_{2}\left(t+1\right)=s_{3}\left(t\right)\\ s_{3}\left(t+1\right)=s_{2}\left(t\right)\vee s_{4}\left(0\right) \end{array}\right.\\ F_{2} & : & \left\{ \begin{array}{c} s_{5}\left(t+1\right)=s_{3}\left(0\right) \end{array}\right.\\ F_{3} & : & \left\{ \begin{array}{c} s_{4}\left(t+1\right)=s_{5}\left(0\right)↔s_{6}\left(t\right)\\ s_{6}\left(t+1\right)=\neg s_{5}\left(0\right) \end{array}\right. \end{array}$$

where *s*_*i*_(0) represents a fixed control value of the input node *v*_*i*_, which is set to 0 or 1 depending on the initial state *S*(0). When the state vectors of *G*_1_, *G*_2_, and *G*_3_ are ordered as (*v*_1_, *v*_2_, *v*_3_, *v*_4_), (*v*_3_, *v*_5_), and (*v*_4_, *v*_5_, *v*_6_), respectively, then the local steady states are detected as follows:

$$\begin{array}{ccc} A_{1} & = & \left\{\langle 0,0,0,0\rangle ,\langle 1,1,1,0\rangle ,\langle 1,1,1,1\rangle \right\}\\ A_{2} & = & \left\{\langle 0,0\rangle ,\langle 1,1\rangle \right\}\\ A_{3} & = & \left\{\langle 0,0,1\rangle ,\langle 0,1,0\rangle \right\} \end{array}$$

where *A*_1_, *A*_2_, and *A*_3_ are the sets of local steady states for *G*_1_, *G*_2_, and *G*_3_, respectively. Firstly, *A*_1_ and *A*_2_ are composed by using the common component *v*_3_ in *G*_1_ and *G*_2_ as the glue between them. As the result, a set of combined local steady states is {⟨0, 0, 0, 0, 0⟩, ⟨1, 1, 1, 0, 1⟩} ordered as (*v*_1_, *v*_2_, *v*_3_, *v*_4_, *v*_5_). The set is then composed with *A*_3_. Finally, two steady states (*i.e.,* {⟨0, 0, 0, 0, 0, 1⟩, ⟨1, 1, 1, 0, 1, 0⟩}) are identified for *G* as the result of composing all the local steady states, which are the same as the ones detected without partitioning.

#### The efficient network partition

In previous sections, we have discussed how we can reliably construct steady states of a given network through partitioning. However, there may exist a number of different partitions available for the given network. In this section, thus, we will discuss how to find the best partition of the network so that the cost to identify all local steady states is minimized. In the proposed approach, we use a SAT solver that is known to be efficient to find steady states of a network. Yet, the complexity of SAT solvers increase exponentially with the number of nodes (*i.e.,* *n*) for BNs with *K* ≥ 2 [24]. Hence, it is computationally more efficient when we partition each block to be smaller in *n* and lower in *K*.

**Fig 3:**
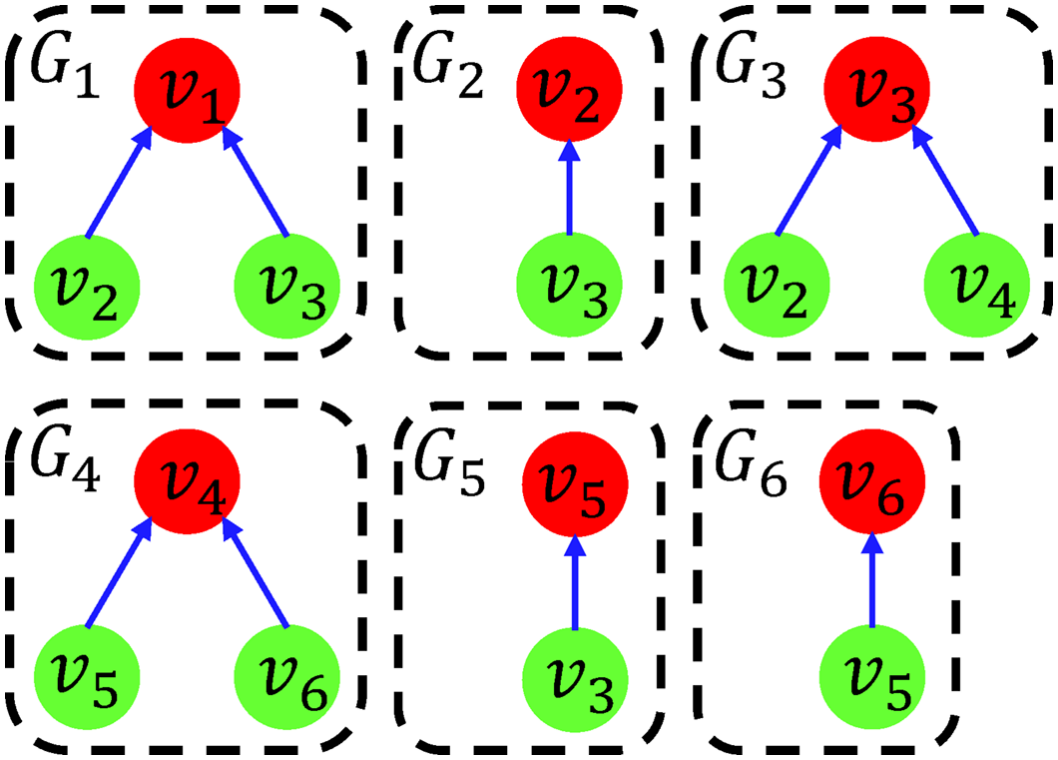
The MEB-based network partition. For the network *G* in Fig 2A, the MEB-based network partition consists of six blocks each of which has a single node (depicted as a red node). Each subnetwork *G*_*i*_ is constructed based on each block. Input nodes are depicted as green nodes.

To utilize a SAT solver in the most efficient way, this paper proposes an optimal partitioning algorithm based on the *minimum essential block* (MEB). Given a BN *G* = ⟨*V*, *F*⟩ with *n* nodes, such a partition can be constructed by dividing *V* into *n* blocks. Thus, each block *V*_*i*_ consists of only a single corresponding node *v*_*i*_. As an example, the MEB-based network partition of the BN *G* of Fig 2A is composed of six blocks such as *V*_1_ = {*v*_1_}, *V*_2_ = {*v*_2_}, *V*_3_ = {*v*_3_}, *V*_4_ = {*v*_4_}, *V*_5_ = {*v*_5_} and *V*_6_ = {*v*_6_}. As shown in Fig 3, six subnetworks are then constructed based on the blocks as described in the previous section. It is worth noting that each subnetwork is the minimum both in *n* and *K* because even if we remove just a single node or an edge from any single subnetwork, the induced alterations of Boolean update rules cause to break the correctness of the algorithm. Therefore, the proposed MEB-based partition guarantees to maximize the performance of a SAT solver while ensuring to determine steady states reliably. A formal proof is given to show that the proposed partition is the best network partition (see S2 Text). The pseudocode of the partitioning algorithm is given in Algorithm 1.

**Algorithm 1** Pseudocode of the network partitioning algorithm

Input: a Boolean network, *G* = ⟨*V*, *F*⟩ with *n* nodes

Output: an optimal network partition, *G*_*min*_ = [*G*_1_, *G*_2_, …, *G*_*n*_]

1: **function** *BEST–PARTITION* (**BN** *G* = ⟨*V*, *F*⟩)

2:  *G*_*min*_ ← [ ]

3:  **for** *i* = 1 to *n* **do**

4:   *V*_*i*_ ← {*v*_*i*_}, *F*_*i*_ ← {*f*_*i*_}

5:   **for** each node *v*_*k*_ ∈ *V* **do**

6:    **if** *v*_*k*_ is an input node of *v*_*i*_ **then**

7:     *V*_*i*_ ← *V*_*i*_∪{*v*_*k*_}

8:    **end if**

9:    *G*_*i*_ ← ⟨*V*_*i*_, *F*_*i*_⟩

10:    *G*_*min*_ ← [*G*_*min*_, *G*_*i*_]

11:   **end for**

12:  **end for**

13:  **return** *G*_*min*_

14: **end function**

#### The efficient steady-state composition algorithm

The overall performance of our algorithm can be further improved when we compose each set of local steady states in a proper order. This is because composing the local steady states in a wrong order may lead to the generation of many redundant intermediate composed steady states which will be discarded later on. If we consider our previous example, we compose local steady states in the order of (*A*_1_, *A*_2_, *A*_3_) resulting in two steady states, {⟨0, 0, 0, 0, 0, 1⟩, ⟨1, 1, 1, 0, 1, 0⟩}. The composition order (*A*_1_, *A*_3_, *A*_2_) can also construct the same steady states, but redundant intermediate composed steady states are generated during the composition. That is, the composition *A*_1_ and *A*_3_ results in {⟨0, 0, 0, 0, 0, 1⟩, ⟨0, 0, 0, 0, 1, 0⟩, ⟨1, 1, 1, 0, 0, 1⟩, ⟨1, 1, 1, 0, 1, 0⟩}, which include two redundant ones. Recall that the composition order (*A*_1_, *A*_2_, *A*_3_) does not generate any redundancy in intermediate composed steady states as shown in the previous example. Thus, the best composition order is the one that generates the least redundant intermediate composed steady states in the course of identification of all steady states. It is, however, generally complicated to find such an optimal composition order in advance since it is often sophisticated to predict which local steady states will be identified in each subnetwork. We thus provide a heuristic algorithm that determines an efficient composition order. The pseudocode of the algorithm is given in Algorithm 2. At each step, the algorithm chooses a subnetwork whose combination with the current combined subnetwork generates the largest number of common nodes between them. The local steady states of the chosen subnetwork are then determined to be composed next. It is worth noting that our composition algorithm composes two local steady states only if the state values of common nodes between them are the same. Thus, it is more likely to filter out redundant intermediate composed steady states when composing with local steady states whose corresponding subnetwork has more common nodes than others at each step. The pseudocode of the composition algorithm is given in Algorithm 3.

**Algorithm 2** Pseudocode for determining an order of composing local steady states

Input: an optimal network partition, *G*_*min*_ = [*G*_1_, *G*_2_, …, *G*_*n*_]

Output: a composition order, *O*_*c*_

1: **function** *DETERMINE–COMPOSITION–ORDER* (**list** *G*_*min*_)

2:  $begin\leftarrow arg\max_{i}\left\{\right|V_{i}\left|\right\}$       /* *V*_*i*_ of *G*_*i*_ ∈ *G*_*min*_ */

3:  *O*_*c*_ ← [*begin*]

4:  *V*_*curr*_ ← *V*_*begin*_

5:  **while** |*O*_*c*_| = *n* **do**

6:   $next\leftarrow arg\max_{i\neq j}\left\{\right|V_{curr}\cap V_{i}\left|\right\}$   /* *j* ∈ *O*_*c*_ and *V*_*i*_ of *G*_*i*_ ∈ *G*_*min*_ */

7:   *V*_*curr*_ ← *V*_*curr*_∪*V*_*next*_

8:   *O*_*c*_ ← [*O*_*c*_, *next*]

9:  **end while**

10:  **return** *O*_*c*_

11: **end function**

**Algorithm 3** Pseudocode of the composition algorithm

Input: an optimal network partition, *G*_*min*_, a list of local steady states, *A*_*loc*_, and a composition order, *O*_*c*_

Output: a set of composed steady states, *A*_*comp*_

1: **function** *ATTRACTOR–COMPOSITION* (**list** *G*_*min*_, **list** *A*_*loc*_, **list** *O*_*c*_)

2:  *s* ← *O*_*c*_ [0]

3:  *A*_*comp*_ ← *A*_*s*_, *V*_*comp*_ ← *V*_*s*_     /* *A*_*s*_ ∈ *A*_*loc*_ and *V*_*s*_ of *G*_*s*_ ∈ *G*_*min*_ */

4:  **for** each *i* ∈ *O*_*c*_ **do**

5:   *C* ← *V*_*comp*_∩*V*_*i*_

6:   *A*_*temp*_ ← ∅

7:   **for** each *a*_*p*_ ∈ *A*_*comp*_ **do**

8:    **for** each *a*_*q*_ ∈ *A*_*i*_ **do**

9:     **if** *SV*(*a*_*p*_, *C*) = *SV*(*a*_*q*_, *C*) **then**

10:      *A*_*temp*_ ← *A*_*temp*_∪{*a*_*p*_ × *a*_*q*_}

11:     **end if**

12:    **end for**

13:   **end for**

14:   *A*_*comp*_ ← *A*_*temp*_

15:   *V*_*comp*_ ← *V*_*comp*_∪*V*_*i*_

16:  **end for**

17:  **return** *A*_*comp*_

18: **end function**

#### Time complexity analysis

Finding steady states of Boolean networks has been proved as NP-hard by Akutsu *et al.* in 1998 [16]. Thus, it is not plausible that the steady-state identification problem can be solved efficiently (*i.e.,* polynomial time) in all cases. However, it is possible to develop algorithms that are fast in the average case, and thus this manuscript studies an algorithm that is efficient in many practical cases.

The proposed algorithm has three main parts as follows: (1) partition the Boolean network, (2) find all local steady states for each subnetwork, and (3) combine the local steady states to the steady states of the overall network. Comparing to (2), the complexity of (1) is negligible as the computational cost increases polynomially with the number of nodes. The worst case time complexity of (1) is *O*(*n* ^2^) because each subnetwork is constructed by checking *n* nodes to find all the control nodes of the corresponding block, where *n* is the size of the network. For (2), on the other hand, the worst case time complexity depends on the performance of a SAT solver, but the time complexity for the (*K* + 1)-SAT problem with *K* ≥ 2 (*i.e.,* corresponds to the BN with *K* ≥ 2) has been proved as NP-complete. Thus, in general, it is very hard to predict which network instances are going to be hard to solve without actually attempting to solve it. The worst case time complexity of (3) is *O*(2^*n*^) if the number of steady states of a BN is 2^n^. However, we may be less interested in such networks as most of biologically meaningful dynamics evolve into relatively small number of steady states. The mean number of attractors of BNs is shown to be proportional to $\sqrt{n}$ [1]. In average cases, the time complexity of (3) is bounded by $O(n^{\frac{3}{2}})$ (for details, see S3 Text).

In summary, the efficiency of the proposed algorithm is mostly determined by the performance of a SAT solver which depends both on *n* and *K*. Since the proposed MEB-based partition minimizes the cost of (2), the proposed algorithm can efficiently detect steady states except in rare cases. The theoretical analysis results are also supported by the simulation results as will be discussed in Results section.

## Results

We have implemented an experimental tool PAD (Partition-based Attractor Detection tool) based on the presented algorithm. The pseudocode of our overall algorithm is given in Algorithm 4. To find the local steady states of each subnetwork, we use a SAT solving algorithm by Dubrova and Teslenko [24] based on MiniSAT SAT solver [31] as shown in lines 4 to 7 of Algorithm 4. PAD is available at http://cps.kaist.ac.kr/∼ckhong/tools/download/PAD.tar.gz.

**Algorithm 4** Pseudocode for the overall steady-state identification algorithm

Input: a Boolean network, *G* = ⟨*V*, *F*⟩ with *n* nodes

Output: a set of steady states, *A*

1: **procedure** MAIN

2:  *G*_*MIN*_ ← *BEST–PARTITION*(*G*)

3:  *A*_*LOC*_ ← [ ]

4:  **for** each *G*_*i*_ ∈ *G*_*MIN*_ **do**

5:   *A*_*i*_ ← MiniSAT(*G*_*i*_)

6:   *A*_*LOC*_ ← [*A*_*LOC*_, *A*_*i*_]

7:  **end for**

8:  *O*_*C*_ ← *DETERMINE–COMPOSITION–ORDER*(*G*_*MIN*_)

9:  *A* ← *ATTRACTOR–COMPOSITION*(*G*_*MIN*_, *A*_*LOC*_, *O*_*C*_)

10: **end procedure**

We analyzed over 3,600 BNs (*e.g.,* real biological process models [9–15], and the *N* − *K* random BNs [3, 40]) to benchmark our method against others. The methods we used for the comparison were those with good computational efficiency: BNS (*i.e.,* the state-of-the-art SAT solver-based attractor detection tool) [24] and ST (*i.e.,* the state-of-the-art partitioning-based attractor detection tool) [25]. It is important to mention that those methods can find not only the fixed points of BN, but also cyclic attractors of the network, which our method is not currently designed to do. There also exist other methods mainly focusing on finding fixed points of the network, but they do not provide their source code or any executable binaries [17, 29]. However, the reported timing of their methods grows exponentially with the number of nodes for BNs with *K* = 3. As we will see later, the timing of our method grows linearly for such networks, so it was not necessary to implement them to include in our benchmark. All the simulations were performed on a machine with Intel(R) Core(TM) i3-2120 CPU@3.30GHz 2-Core with 4GB memory running Ubuntu 12.04.

### Results for Boolean networks models of real biological processes

We tested the proposed and other methods on seven Boolean network models of real biological processes: control of flower morphogenesis in the mammalian cell cycle regulation [10], fission yeast cell cycle regulation [14], budding yeast cell cycle regulation [13], T-helper cell differentiation [11], T-cell receptor signaling pathway analysis [12], Drosophila melanogaster segment polarity genes expression patterns prediction [15], and T-cell large granular lymphocyte (T-LGL) leukemia signaling [9].

The simulation results are summarized in Table 1. Columns 1, 2, 3 and 4 show the name of the model, the number of nodes (*i.e.,* *n*), the maximum indegree (*i.e.,* *K*) and the number of fixed points computed, respectively. In columns 5, 6 and 7, we show the runtime of BNS, ST, and PAD, respectively. As shown in Table 1, the models are all small, but have high *K*. The results indicate that the proposed tool performs better than BNS by 2.91 times, and ST by 3.27 times in average. Interestingly, BNS is faster than ST although ST partitions the given model to several blocks, and applies a SAT solver to each block while BNS applies a SAT solver without partitioning. A possible reason is that ST needs to compute strongly connected components (SCC) to partition the given BN based on those, and such overhead caused not to improve the performance of ST in the small networks. The partitioning method of the proposed algorithm is simpler than the one of ST, and also minimizes both *n* and *K* of each block. Therefore, the computational advantage gained by the proposed partition can dominate the overhead of the partitioning procedure, and so PAD outperforms both BNS and ST even in the small networks.

**Table 1:** Simulation results for detecting steady states from real Boolean networks.

Models	# of nodes	K	# of steady states	Runtime (sec)		
				BNS [24]	ST [25]	PAD (Ours)
Mammalian cell [10]	10	6	1	0.003	0.006	0.001
Fission yeast [14]	10	6	13	0.003	0.004	0.003
Budding yeast [13]	12	6	7	0.009	0.009	0.005
T-helper cell [11]	23	5	3	0.003	0.004	0.001
T-cell receptor [12]	40	5	8	0.018	0.021	0.009
Drosophila melanogaster [15]	52	6	7	0.027	0.043	0.019
T-cell LGL leukemia signaling [9]	60	7	1	1.371	0.877	0.168

### Results for random Boolean networks

The models in Table 1 consist of several tens of genes only, which is a typical size used in most published models today. It is known, however, that the number of genes involved in many processes of biological interest exceeds the size of these models by at least an order of magnitude [28]. The number of functionally relevant interactions between genes of these models, representing the links, is expected to be much more complex (*i.e.,* higher *K*) [25]. In order to evaluate how well our algorithm can scale up on larger and more complex examples, a large set of BN with different *K*s are randomly generated. We use the *BoolNet* package [41] in the *R* environment [42] to generate *N* − *K* random BNs. To generate more biologically realistic random BNs, the parameters of *generateRandomNKNetwork* function are set to *K* = 2 to 5 and *topology* = *scale_free*, where *K* and *topology* represent parameters for the maximum indegree and the network structure of the BN, respectively. Note that many real biological networks have the scale-free property such that *K* of these models greatly exceeds the average indegree [34, 43, 44].

**Fig 4:**
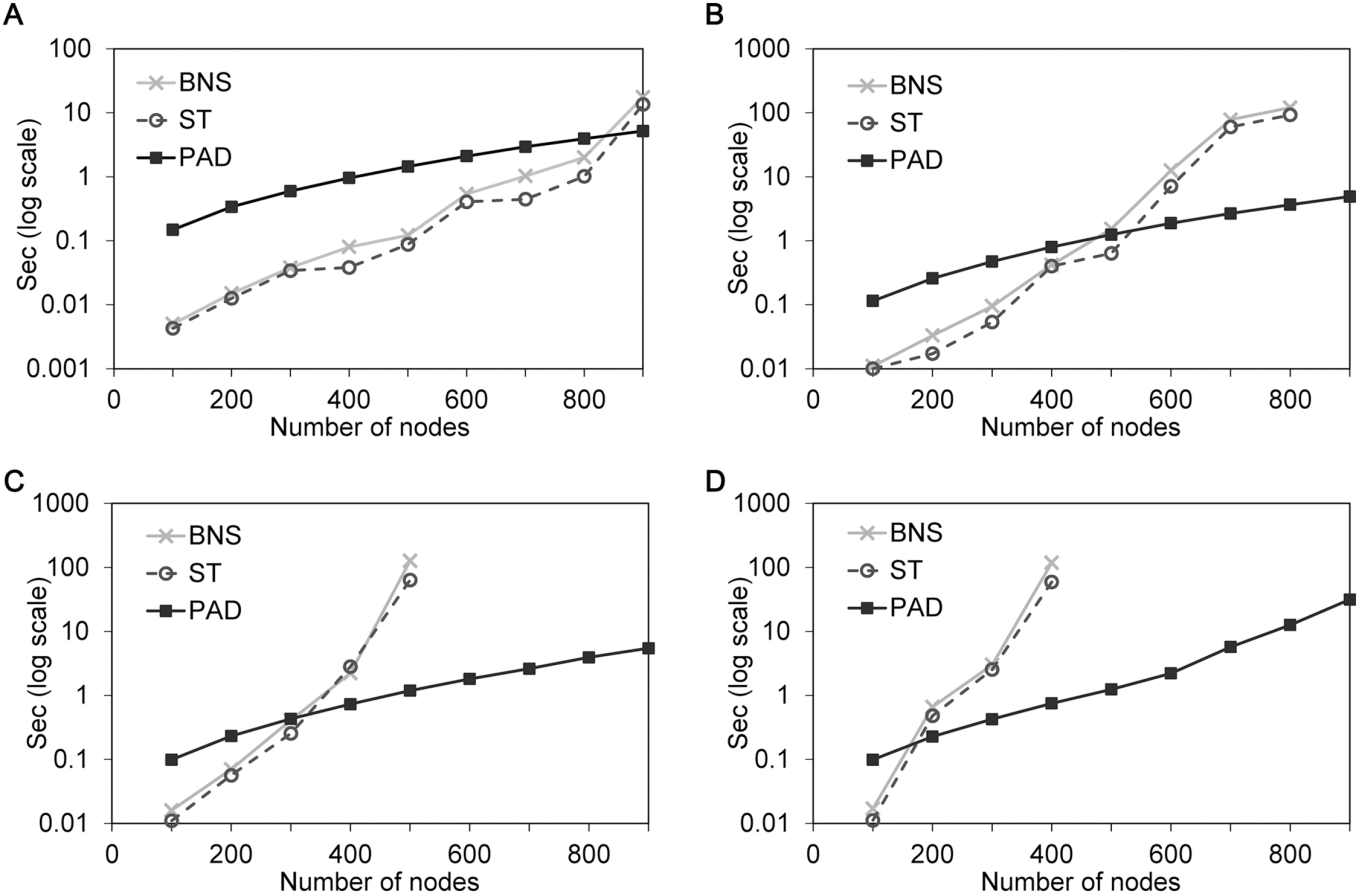
Runtime comparisions of algorithms. The simulation results for 3,600 randomly generated Boolean networks (BN) with maximum indegree *K*. Each dot is the median value computed for 100 networks. A: Median runtime of algorithms for BN with *K* = 2. B: Median runtime of algorithms for BN with *K* = 3. C: Median runtime of algorithms for BN with *K* = 4. D: Median runtime of algorithms for BN with *K* = 5.

In the first simulation, we applied the algorithms to compute the median runtime per steady state for 3,600 randomly generated BNs of sizes between *n* = 100 and *n* = 900 nodes with *K* = 2 to *K* = 5. The timeout is set to 1000 seconds, and the results only include the data within the timeout as shown in Fig 4. Each dot is computed for 100 networks. As we can see in Fig 4B–4D, the median runtime per steady state for BNS and ST grows exponentially with *n*. We can also see that all timings grow much faster for networks with increasing *K*. Interestingly, BNS performs almost the same as ST for networks with *K* = 4 or 5. We consider that the partitioning method implemented in ST focuses on reducing costs to find steady states in networks with relatively low *K*. The timing of our algorithm, however, grows linearly with *n* even for networks with high *K* = 5.

On the other hand, PAD performs slightly worse than other tools when networks have relatively low *K* and small *n*, as we can see in Fig 4. We consider that the results may be due to the fact that the proposed partitioning method is optimized to handle mainly large networks with high *K*. But, at the same time, more number of subnetworks can be constructed, which may induce an overhead during composing local steady states of them. Meanwhile, a SAT solver itself shows good scalability for networks with up to *n* = 800 nodes when *K* = 2 as shown in Fig 4A. Thus, we account for that the proposed fine-grained partition is over-optimized for such BNs and caused the overhead in the procedure of composition. The proposed partitioning method, however, enables each subnetwork to maintain small size and simple structure even for large BNs with high *K*s. It is worth noting that the cost to compute steady states of other methods grows exponentially with *n* and *K*. This is because their units of partitioning (*i.e.,* the given BN itself in BNS and the strongly connected component in ST) are still too large and complex for a SAT solver to compute steady states efficiently. Compared to those timings, the aforementioned overhead of our method is negligible. Therefore, the proposed fine-grained partition enables our method to be much more scalable than others.

**Fig 5:**
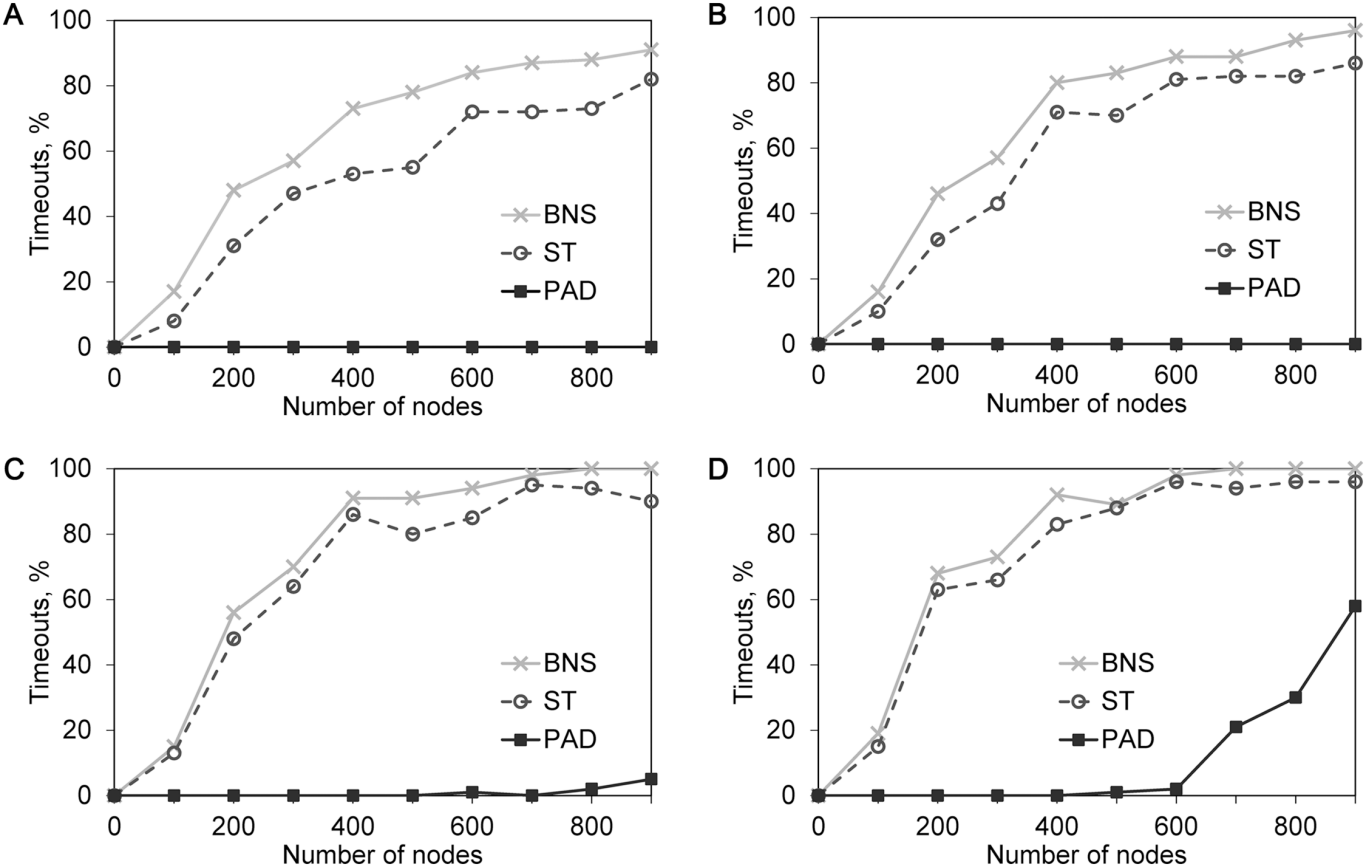
Scalability comparisions of algorithms. The simulation results for 3,600 randomly generated Boolean networks (BN) with maximum indegree *K*. Each dot is the percentage of timeouts computed for 100 networks. A: Timeout percentage of algorithms for BNs with *K* = 2. B: Timeout percentage of algorithms for BNs with *K* = 3. C: Timeout percentage of algorithms for BNs with *K* = 4. D: Timeout percentage of algorithms for BNs with *K* = 5.

In the second simulation, we compared the presented algorithm to other algorithms in terms of the number of successful terminations within 60 sec. We used the same random BNs as the ones generated in the first simulation. The results are shown in Fig 5. Each dot is computed for 100 networks. As we can see, for BNS the number of successful termination rapidly approaches 0 for *n* > 200. In comparison to BNS, the number of successful termination of ST approaches 0 slightly slower than BNS, but it rapidly reaches 0 when *K* increases. On the other hand, PAD is able to handle 40 percent of networks even for *n* = 900 and *K* = 5. The results show that increasing *K* has a dramatic effect on the sizes of feasible networks that can be studied. Especially for BNS and ST, further increasing *K* will have a much more dramatic effect. We consider that the larger (*i.e.,* *n* ≥ 1000) and more complex (*i.e.,* *K* ≥ 5) BNs would be the trend for real biological process models [25, 45, 46]. We claim that PAD would be more useful in those BNs because of the scalability although our method is not currently equipped to find cyclic attractors.

## Discussion

Finding steady states is one of the key problems in the analysis of Boolean network models of biological processes. In this manuscript, we presented an efficient algorithm for the determination of steady states based on partitioning and a SAT solver. Our method ensures each block of the partition to be the minimal both in size (*i.e.,* *n*) and the maximum indegree (*i.e.,* *K*) such that maximizes the performance of a SAT solver to identify local steady states. The correctness of our proposed algorithm is also formally proved. We have used two types of networks for benchmark: published models for real biological processes, and randomly generated scale-free networks. Our simulation results show that the inherent overhead (*i.e.,* partitioning and composing costs) of our method is compensated by the efficiency of the provided partition, and thus our implementation tool, PAD, shows good scalability even on large random networks with high *K*s.

In 2007, Naldi *et al.* [20] proposed a steady-state analysis algorithm by using decision diagrams (DDs) each of which is constructed based on a Boolean function associated with a node. It is worth noting that each DD similarly corresponds to each subnetwork of our approach. Each DD (*i.e.,* local DD) is then composed in a pairwise manner, and the steady states of the given network are determined by checking the finally composed DD (*i.e.,* global DD). In the course of the composition, redundant intermediate composed local DDs can be temporarily generated, which will be discarded later on. Depending on the order of DD compositions, such redundant DDs can be significantly increased, thus making the intermediate combined DD become too memory-consuming. Such redundancy may be substantially reduced when composed in a good order, but they do not provide how to determine such an order. Thus, this limits the Naldi *et al.*’s algorithm [20] to deal with only small (*i.e.,* about a hundred nodes [47, 48]) Boolean networks like other DD-based algorithms [18–21]. Our partitioning method is similar to that of Naldi *et al.*’s study [20]. However, our steady-state detection algorithm is different from the Naldi *et al.*’s in two aspects as follows [20]: (1) instead of using DD, we use a SAT solver to analyze each subnetwork. (2) in contrast to Naldi *et al.*’s study [20], we provide an efficient composition order that reduces redundancy in intermediate steady states to be generated during composing local steady states. Thus, it results in a significantly smaller size of combined local steady states than that of its corresponding combined local DD as the composition steps continue. The above differences enable our composition method to have much smaller composition cost than the Naldi *et al.*’s [20]. Hence, this contributes to the enhanced scalability of our steady-state identification method although partitioning methods are similar. Here it should be noted that Naldi *et al.* provide a user-friendly GUI tool, GINsim, and it can handle multiple-valued networks too [20].

Zhao *et al.* [33] and Guo *et al.* [25] also exploited network partitioning methods to identify steady states of Boolean networks efficiently. As shown in the simulation results, however, the execution time of their methods grows exponentially with *n* and *K*. Specifically, for scale-free random networks with *K* = 5, they seemed not to terminate at all as *n* increases. The reason is that the size of the largest SCC is too large to be analyzed within a reasonable timing for such networks with relatively high *K*. In contrast to the results of Zhao *et al.* [33] and Guo *et al.* [25], PAD is scalable for the scale-free random Boolean networks up to several hundreds of nodes by favor of the MEB-based partition. The published Boolean network models analyzed in this manuscript have high *K*, but consist of several tens on nodes only. However, the size of published models is growing, and the largest SCC of such models connects the vast majority of nodes [35–39]. It is also reported that the maximum indegree of published models is orders of magnitude higher than the average indegree [34]. Thus, we believe that the demonstrated scalability for finding steady states will be a key functionality in any systems biology toolkit as more published large and scale-free networks become available.

Alongside the partitioning methods, network reduction methods are another direction of research in steady-state analysis [49–55]. For example, Zanudo and Albert [54] recently proposed a method that uses network motifs (*i.e.,* subgraphs) to reduce the given networks. Then, the reduced networks are analyzed to find not only the steady states, but also cyclic attractors. By means of the network reduction method, they achieved good scalability up to 200-node BNs with indegree two. But, compared to our method, such network reduction based algorithms themselves are not scalable enough to handle large networks. However, it is worth noting that network reduction methods are beneficial when applied orthogonally to the existing steady-state identification methods to improve them. As an example, in 2014, Veliz-Cuba *et al.* [55] extended a process algebra based steady state finding algorithm by incorporating a network reduction. Once the reduced network is constructed, they can efficiently compute its steady states by applying the computational algebra technique. To take a lesson from them [55], we will consider to apply network reduction methods orthogonally to our algorithm in the future. We believe that the hybrid approach will give us a better chance to tackle challenges in identifying steady states, which is still unsolved in general. Another direction of our future work is to extend our method to determine all attractors including cyclic attractors.

## Supporting Information

S1 Text Correctness proof. The file presents the correctness proof of our steady-state detection algorithm. Open with your favorite pdf reader, *e.g.,* Adobe Reader.
(PDF)

S2 Text Optimality proof. The file presents the optimality proof of our partitioning algorithm. Open with your favorite pdf reader, *e.g.,* Adobe Reader.
(PDF)

S3 Text Analysis of the average time complexity of our composition algorithm. The file presents the analysis of the average time complexity of our composition algorithm. Open with your favorite pdf reader, *e.g.,* Adobe Reader.
(PDF)
