# Supplementary material for: An Efficient Steady-State Analysis Method for Large Boolean Networks with High Maximum Node Connectivity
Source: PLoS One. 2015 Dec 30;10(12):e0145734. doi: 10.1371/journal.pone.0145734 (PMC4700995; doi:10.1371/journal.pone.0145734)
Supplement: S1 Text — The file presents the correctness proof of our steady-state detection algorithm. Open with your favorite pdf reader, e.g., Adobe Reader. (PDF) [file pone.0145734.s001.pdf]

Correctness proof

Given a Boolean network $G = \langle V, F \rangle$ with a node set $V = \{v_1, v_2, ..., v_n\}$ and its Boolean update rule $F = \{f_1, f_2, ..., f_n\}$, the proposed algorithm divides G into G' with m subnetworks as follows:

$$G' = \{G_1, G_2, ..., G_m\} \tag{1}$$

$$G_i = \langle V_i \cup C_i, F_i \rangle \tag{2}$$

$$V = V_1 \cup V_2 \cup ... \cup V_m \tag{3}$$

$$C_i = \{v_k | v_k \text{ is an input node of } V_i\} \tag{4}$$

$$F_i = \{f_k | f_k \text{ is a Boolean update rule of } v_k \in V_i\} \cup F_{C_i} \tag{5}$$

$$F_{C_i} = \{f'_k | f'_k = 0 \text{ or } 1 \text{ where } v_k \in C_i\} \tag{6}$$

where V_i is a proper subset of V. Here, we provide a formal correctness proof for the proposed steady state detection algorithm through partitioning. To this end, we define A_G as the set of steady states identified directly from the original Boolean network G, and $A_{G'}$ as the steady states computed by the proposed algorithm. The condition $A_G = A_{G'}$ should be satisfied to prove the correctness of the proposed algorithm.

Theorem. A_G is equal to $A_{G'}$.

Proof. ($\Longrightarrow$) Firstly, we prove that the condition, if $\alpha \in A_G$ then $\alpha \in A_{G'}$, always holds. Suppose that there exists such a state vector α, which satisfies $F(\alpha) = \alpha$. For each subnetwork $G_i \in G'$, local steady states are analyzed by considering all the possible combinations of value vectors of C_i including $SV(\alpha, C_i)$. Here, we define $SV(\alpha, C_i)$ as a subvector extracted from the vector α according to the target output C_i. By equations (5) and (6), local steady states of each subnetwork G_i include the state vector α_i that satisfies $F_i(\alpha_i) = \alpha_i = SV(\alpha, V_i)$. Therefore, combining such α_i for all subnetworks reliably constructs α.

($\Longleftarrow$) Conversely, we prove that the condition, if $\alpha \in A_{G'}$ then $\alpha \in A_G$ is always true. Similarly as above, such a state vector α is constructed by combining all α_is each of which satisfies $F_i(\alpha_i) = \alpha_i$. By equations (5) and (6), $F|V_i(\alpha_i) = \alpha_i = SV(\alpha, V_i)$ is satisfied, where $F|V_i$ is the restriction of the update rule F by choosing update rules of V_i. Thus, steady states of the original network G with the update rule F also include α. □
