# Supplementary material for: An Efficient Steady-State Analysis Method for Large Boolean Networks with High Maximum Node Connectivity
Source: PLoS One. 2015 Dec 30;10(12):e0145734. doi: 10.1371/journal.pone.0145734 (PMC4700995; doi:10.1371/journal.pone.0145734)
Supplement: S2 Text — The file presents the optimality proof of our partitioning algorithm. Open with your favorite pdf reader, e.g., Adobe Reader. (PDF) [file pone.0145734.s002.pdf]

Optimality proof

Theorem. If the given network G is divided into a subnetwork-set G_{min} by using our MEB-based partitioning method, each subnetwork of G_{min} is the smallest in terms of the correctness of our steady-state detection algorithm.

Proof. We prove by contradiction. Suppose that there exists a subnetwork $G_k \in G_{min}$ which can be still divided into smaller subnetworks. Let us remove v_p and its corresponding edges from G_k, and call such a subnetwork and a subnetwork-set as G_k^- and G_{min}^-, respectively. Here, G_k^- is smaller than G_k, but the correctness of our steady-state detection algorithm is, however, no longer guaranteed in G_{min}^-. This is because the Boolean update rules of nodes related to v_p are altered such that local steady states of G_k^- cannot include the correct ones to construct the steady states of overall network. This contradicts the assumption that the subnetwork $G_k \in G_{min}$ can be divided into smaller subnetworks. Thus, the set G_{min} guarantees each subnetwork to be the smallest both in the size and the maximum indegree while ensuring the correctness of our steady-state identification algorithm. □
