# Supplementary material for: An Efficient Steady-State Analysis Method for Large Boolean Networks with High Maximum Node Connectivity
Source: PLoS One. 2015 Dec 30;10(12):e0145734. doi: 10.1371/journal.pone.0145734 (PMC4700995; doi:10.1371/journal.pone.0145734)
Supplement: S3 Text — The file presents the analysis of the average time complexity of our composition algorithm. Open with your favorite pdf reader, e.g., Adobe Reader. (PDF) [file pone.0145734.s003.pdf]

Analysis of the average time complexity of our composition algorithm

We assume that the given Boolean network $G = \langle V, F \rangle$ with n nodes is divided into n subnetworks (*i.e.*, $G_1, G_2, ..., G_n$, where $G_i = \langle V_i, F_i \rangle$). We denote A_i and $|A_i|$ as local steady states detected from G_i and the size of A_i, respectively. The average number of local steady states is shown to be proportional to $\sqrt{|V_i|}$, where $|V_i|$ is the size of V_i [1]. The average cost of composing A_1 and A_2 is thus $\sqrt{|V_1|} \times \sqrt{|V_2|}$. Then, the combined local steady states (denote as A_{12}) are composed with A_3, and the current total cost of composition including the cost of composing A_{12} and A_3 is thus

$$\sqrt{|V_1| \times |V_2|} + \sqrt{|A_{12}| \times |V_3|}. \tag{1}$$

Since A_{12} is the same as the steady-state analysis result of the combined subnetwork of G_1 and G_2 (denote as $G_{12} = \langle V_1 \cup V_2, F_1 \cup F_2 \rangle$), $|A_{12}|$ is equal to $\sqrt{|V_1 \cup V_2|}$. Therefore,

$$\sqrt{|V_1| \times |V_2|} + \sqrt{|A_{12}| \times |V_3|} = \sqrt{|V_1| \times |V_2|} + \sqrt{|V_1 \cup V_2| \times |V_3|}. \tag{2}$$

In this manner, the total cost of composing A_1, A_2, ..., and A_n is

$$\sqrt{|V_1| \times |V_2|} + \sqrt{|V_1 \cup V_2| \times |V_3|} + ... + \sqrt{|V_1 \cup V_2 \cup ... \cup V_{n-1}| \times |V_n|}. \tag{3}$$

Since $|V_1 \cup V_2 \cup ... \cup V_{n-1}| < n$, the total composition cost is bound by

$$n \times \sqrt{n \times |V_{max}|} \tag{4}$$

where $|V_{max}| = \max\{|V_i|\}$. Thus, the average time complexity of our composition algorithm is $O(n^{\frac{3}{2}})$.

References

1. de Jong H. Modeling and Simulation of Genetic Regulatory Systems: A Literature Review. Journal of Computational Biology. 2002;9(1):67103. Available from: http://dx.doi.org/10.1089/10665270252833208.
